# Emotion Regulation and Emotion Work: Two Sides of the Same Coin?

**DOI:** 10.3389/fpsyg.2012.00496

**Published:** 2012-11-16

**Authors:** Christian von Scheve

**Affiliations:** ^1^Department of Sociology and Cluster of Excellence “Languages of Emotion,” Freie Universität BerlinBerlin, Germany

**Keywords:** emotion regulation, emotion work, deep acting, feeling rules, emotion norms

## Abstract

This contribution links psychological models of emotion regulation to sociological accounts of emotion work to demonstrate the extent to which emotion regulation is systematically shaped by culture and society. I first discuss a well-established two-factor process model of emotion regulation and argue that a substantial proportion of emotion regulatory goals are derived from emotion norms. In contrast to universal emotion values and hedonic preferences, emotion norms are highly specific to social situations and institutional contexts. This specificity is determined by social cognitive processes of categorization and guided by framing rules. Second, I argue that the possibilities for antecedent-focused regulation, in particular situation selection and modification, are not arbitrarily available to individuals. Instead, they depend on economic, cultural, and social resources. I suggest that the systematic and unequal distribution of these resources in society leads to discernible patterns of emotion and emotion regulation across groups of individuals.

## Introduction

Emotion research over the past decades has increasingly portrayed emotions as adaptive responses to evolutionary demands, firmly rooted in biological and psychological response mechanisms. Studies have consistently emphasized their functions in individual (Levenson, 1999) and social or cultural terms (Keltner and Haidt, 1999; Thoits, 2004). As such, emotions have been shown to contribute to cognitive processing, decision-making, and memory formation as well as to the emergence of social bonds and relationships, the coordination of social action, and the maintenance of social order. But this has not always been the case. From the Greek philosophers to the Scottish moralists and the modern counseling literature, passions and emotions have often been considered as disturbing and irritating occurrences in human life, in particular in domains requiring calm deliberation and rational thought. Therefore, and although emotions are ubiquitous to human affairs, the ability to control and manage one’s emotions has become a key driving force of civilization and a hallmark of modern societies (Elias, 1939/1978). We aim at not forgetting ourselves when faced with indignity, at still being courteous at some boring dinner party, or at getting rid of that gloomy feeling. It thus seems as if there occasionally was something “undesirable” or even potentially “dangerous” to emotions, both in view of social bonds and relationships as well as with respect to subjective experience and individual behavior.

This suggests that emotions’ evolutionary founded “wisdom of the ages” (Lazarus, 1991, p. 820) is not indeterminately appropriate and may in fact jeopardize one’s goals and social integration (Gross et al., 2006). Clearly, changing environmental conditions alter emotion’s response contingencies, and not all emotional reactions are always adaptive and individually or socially beneficial, in particular in contemporary societies. Or, as Gross (1999, p. 558) put it: “Physical and social environments have changed out of all recognition from those that shaped our emotions, and technological advances have dramatically magnified the consequences that our emotional responses may have for ourselves and others. An irritable swipe that once scarcely raised a welt, is now translated with the greatest ease into a fatal car accident or gun-related homicide.” It seems that the basic architecture and some of the mechanisms that elicit emotions have remained largely unchanged over the course of evolution, whereas the social and cultural environments have changed dramatically. Part of this mismatch obviously gives rise to the desire to alter and manage existing emotional states.

Despite their evolutionary roots, emotions have proven to be highly adaptive to dominant cultural and social conditions (Hochschild, 1983; Thoits, 2004; Boiger and Mesquita, 2012). Norms, rules, values, and the social practices through which they are learned and internalized all contribute to the cultural shaping of emotion. From this perspective, it can be argued that emotions are always regulated in a longer-term understanding and in a sense that they are “calibrated” to culture and society (Vandekerckhove et al., 2008; von Scheve, 2012).

This “one-factor” view of emotion regulation holds that “emotion and regulation are one” (Kappas, 2011), and that the regulation of emotion is not limited to an actual emotion episode, but rather extends throughout ontogenetic development. In this vein, some have argued that “emotion and emotion-control are part and parcel of the same processes and any scientifically viable theory of emotion must also be a theory of emotion-control” (Kappas, 2008, p. 15; see also Campos et al., 2004; Kappas, 2011). Much like sociological and psychological one-factor views highlight the importance of the social and cultural embeddedness for emotion regulation, recent biological and physiological accounts emphasize the importance of individuals’ ecological embeddedness. Beckes and Coan (2011), for example, argue that social proximity and interaction should not only be taken into account as indicators prompting (intentional) emotion regulation in an encounter, but also as referents of the degree of embeddedness into social networks that signal a “baseline” of social integration, which in turn renders the organism more or less susceptible to emotional arousal.

Although such longer-term regulatory processes tend to operate implicitly and automatically (Mauss et al., 2008), they also clearly include instances in which existing emotions are deliberately altered to meet certain social or cultural requirements. Mostly, these instances also contribute to the adaptation and fine-tuning of emotion to a socio-cultural context, although they equally well serve individual goals. This regulation of an existing emotional state – such as when getting rid of one’s anger or amplifying a good mood to outward joy – corresponds to the analytical “two-factor” perspective on emotion regulation (see Campos et al., 2004, p. 377). This perspective assumes one set of processes related to the elicitation of emotion (first factor) and a second set directed at the regulation or control of an existing emotion (second factor).

Based on these premises, issues in the regulation and management of emotion have become a lively field of inquiry in the social and behavioral sciences. Traditionally, different disciplines have been concerned with different aspects of emotion regulation. The behavioral sciences, above all psychology, have developed advanced micro-level models that focus on the individual processes and mechanisms underlying emotion regulation. In the social sciences, in particular in sociology, research is dominated by macro-level accounts of social norms and rules to which individuals refer in modulating emotional experience and expression.

In this contribution, I ask how psychological two-factor models of emotion regulation can be extended to accommodate “macro-level” social and cultural influences on the regulation of emotion, as they have been documented by sociological and cultural emotion theories. Often, research on the influence of culture and society on emotion regulation has focused primarily on one-factor models and long-term influences (Denzin, 1990; Thoits, 1990). Here, I will primarily take a two-factor perspective to highlight the impact of the social world from the standpoint of methodological individualism or situationalism. The aims of the article therefore are to link both perspectives to achieve a better understanding of the social embeddedness of emotion regulation, to show how psychological and social-cultural processes interact in emotion regulation, and to pave the way for an exchange between disciplines that have mostly attended to the regulation of emotion in disparate ways.

I will first briefly review Gross’s (1999) well-established process model of emotion regulation and highlight key processes that are particularly susceptible to social influences or even require information from the social environment. In a second step, I discuss sociological approaches to emotion management and regulation, in particular the widely adopted notion of “emotion work.” Here, I will emphasize the role of social norms and different institutional settings to which they belong. In a third step, I will frame these determinants of emotion regulation as emotion regulatory goals in Gross’s process model. Moreover, I will argue that the possibilities of antecedent-focused emotion regulation, in particular *situation selection* and *situation modification* (Gross and Barrett, 2011), are not arbitrarily available to individuals. Instead, the ability to select and modify situations depends on different kinds of resources, in particular economic, cultural, and social resources, which affect regulatory effort.

## Individual Processes in Emotion Regulation

Although two-factor theories of emotion regulation differ in their details, most of those taking an individual or dyadic perspective converge in their definitions and understandings of what emotion regulation is. According to Gross, “emotion regulation refers to the processes by which individuals influence which emotions they have, when they have them, and how they experience and express these emotions. Emotion regulatory processes may be automatic or controlled, conscious or unconscious, and may have their effects at one or more points in the emotion generative process” (Gross, 1998, p. 275; italics omitted).

According to this view, fully understanding emotion regulation requires a compatible definition of what an emotion is. Although this is constantly debated in emotion research (e.g., Kappas, 2002), recent psychological and sociological approaches converge on a *componential* definition of emotion. In this light, emotions are elicited by the evaluation or appraisal of (internal or external) cues that are in one or another way relevant for the individual. These evaluations then trigger a pattern of coordinated responses tendencies that are supposed to facilitate adaptive behavior. These responses tendencies form the basis of an emotion episode and include experiential, cognitive, behavioral, and physiological components (Gross, 1999; Scherer, 2005; Thoits, 2007).

Emotion regulation in principle extends to most of these components and involves changes in expressive behavior, subjective feeling, or physiological responses (cf. Gross, 1999, p. 557; Gross, 2002, p. 282). This definition encompasses not only negative emotions but also the processes whereby emotions are strengthened, maintained, or weakened, regardless of their valence. It also allows to make a distinction between the conscious and intentional regulation of an emotion on the one hand, such as changing the topic of conversation that is getting annoying, and automatic and unconscious regulation on the other hand, such as always appearing to be grateful when receiving a present, even if the present comes close to an offense. However, this perspective on emotion regulation largely excludes conceptions of emotion regulation that refer to the regulatory *functions* of emotions. In these cases, emotion regulation is used to indicate emotions capacity to regulate some other mental or physiological process, for example perception, memory retrieval, or decision-making (e.g., Baumeister et al., 2007). Also, this understanding highlights the intrapersonal aspects of emotion regulation and excludes the regulation of other individuals’ emotions in social interaction, which is often referred to as “emotion management” (Lively, 2010).

**Figure 1:**
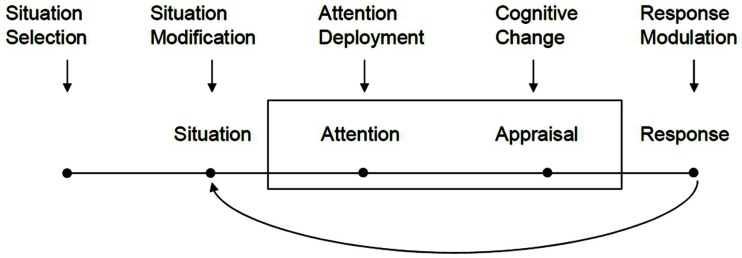
**Basic process model of emotion regulation reproduced from Gross and Barrett (2011, p. 12)**.

A well-established psychological theory of emotion regulation that closely aligns with the above definitions is Gross’s model of emotion regulation (e.g., Gross, 1999). Understanding emotion regulation as a process, the model assumes the existence of an emotion episode or situation and identifies five distinct stages at which this episode can be modulated. These stages can be further differentiated in “antecedent” (aiming at changes in the antecedents of an emotion) and “response” oriented regulation (changing the emotional response-components). Figure 1 represents the basic structure of the model.

The model is organized along a time axis representing analytically distinct phases in the elicitation of an emotion. Antecedent-oriented regulation kicks in at an early stage in the emotion generative process and focuses on changing the situational circumstances that give rise to an emotion in the very first place. Habitually avoiding unsuitable or unpleasant topics of conversation is an example of this type of regulation or dismissing an employe who is a frequent cause of anger. Response-oriented regulation, on the other hand, refers to strategies employed when an emotion, including most of its response-components, have already manifested. These strategies are directed at changing the effects or immediate consequences of an emotion, such as the suppression of a facial expression.

Antecedent-oriented regulation encompasses several possibilities: the selection and modification of the emotionally relevant situation, the deployment of attention, and the (cognitive) interpretation or appraisal of the present situation (cf. Gross, 1998, 2002, p. 282). Situation selection as the first possible step of antecedent-oriented regulation aims at seeking, creating, or avoiding situations in which actors expect certain emotions to occur, either based on experience or actual exposure. If actors are unable to actively seek or avoid a situation, the modification of a situation still allows changing its emotionally relevant constituents in such a way that a desired emotion is experienced or an undesired is avoided.

Actors can also change attentional deployment and focus on selected aspects of a situation or actively disregard others to regulate emotions. Ignorance of certain facts or persons is a well-known strategy in this regard. A closely related approach is the active and deliberate modification of one’s (cognitive) evaluation of the situation or of a certain aspect thereof. This “reappraisal” or re-interpretation involves the re-framing of a situation and the re-examination of the preceding appraisal that elicited the actual emotion state (e.g., Urry, 2009). Such reappraisals or cognitive changes imbue a situation with a meaning different from an originally assigned meaning and consequently give rise to changes in the related emotion (Gross, 2002).

Response-oriented regulation taps changes in the various consequences or components of an emotion. Good examples of this type of regulation are the suppression or evocation of a facial expression or the regulation of physiological reactions, such as efforts to calm down or to curb motor reactions. The key difference between both kinds of regulatory effort is that antecedent-focused regulation usually aims at changing or producing an emotional reaction in its entirety or simply at disposing an existing emotion. Response-focused regulation rather aims at dealing with the consequences of an emotion and usually does not target the entire emotional response (although, of course, one could argue that getting rid of the phenomenal component of an emotion is pretty much the same as getting rid of an emotion in its entirety).

## Emotion Regulation Goals

Given the strategies of emotion regulation illustrated above, an interesting question is why and to what ends people engage in emotion regulation at all. One of the most straightforward and empirically substantiated answers is for hedonic pleasure (Rusting and Larsen, 1995; Vastfjall et al., 2001). However, there is a broad array of other things that people value that are not necessarily associated with pleasure, for instance social conformity, health, or utility (e.g., Higgins, 2006; Tamir, 2009), that might prompt emotion regulatory behavior. Nevertheless, accounts of pleasure and pain as motives of emotion regulation have dominated the literature (Tamir and Mauss, 2011) whereas the role of values and goals has been much more at the heart of self-regulation than emotion-regulation research.

Generally, understanding the goals and motives of emotion regulation also involves understanding the things people value in affective and emotional terms. The focus on hedonic pleasure in recent research is rooted in the nature of emotions as intrinsically pleasant or unpleasant experiences. In this vein, the standard view holds that people value and aim at seeking pleasant emotions and at avoiding unpleasant ones. However, it is not completely clear what makes a pleasant emotion pleasant. Tamir (2009) has questioned the assumption that people always want to feel “pleasant” emotions. Instead, she highlights the role of short-term and long-term benefits and argues that “unpleasant” emotions, such as anger, are often sought to aide long-term goal attainment. Similarly, research on esthetic emotions indicates that allegedly unpleasant emotions, for example intense sadness, are often actively sought and enjoyed (e.g., Oliver and Woolley, 2010). Clearly, the regulation of emotion is closely tied to the feelings that are preferred and valued by a person. These values and preferences for certain emotions in certain situations develop in social and cultural contexts and are internalized during the course of socialization. Studies have demonstrated that there are marked differences between cultures in view of which emotions are valued and which are not (Eid and Diener, 2001). These studies, however, are usually based on an understanding of “culture” as primarily depending on geopolitical location and language, such that one of the most investigated differences is that between supposedly “collectivist” Asian and “individualist” Western groups. Also, research has demonstrated robust cross-cultural differences in the valuation of what Tsai et al. (2006) term “ideal affect.” Ideal affect refers to the affective states that people value, prefer, and ideally want to feel. It is at the core of what a “good feeling” actually is (Tsai, 2007).

From a sociological point of view, the question of the role of values and goals is interesting because it warrants the assumption that there are patterns and regularities in emotion regulation across large numbers of individuals. Departing from cross-cultural psychological approaches, however, sociology is more interested in cultural differences *within* societies, for example across social classes or in different institutional settings. Therefore, the following section discusses select sociological views on the regulation and social control of emotion that put social norms and institutional settings at the forefront.

## Social and Cultural Processes in Emotion Regulation

Although many sociological accounts of emotion regulation would stress the general importance of a one-factor account, a prominent line of inquiry focuses on the regulation of emotion that is in principle in accordance with the processes outlined by psychological research. Clearly, these accounts often emphasize the importance of social practices, symbolic interaction, and normative orders over individual processes (Thoits, 2004), but they still rely on assumptions on how the two interface with one another.

Interesting in this regard, Hochschild (1979) promoted two possible approaches to the “social ordering of emotive experience.” The first is based on the analysis of the “social factors that induce or stimulate primary […] emotions,” whereas the second one is “to study secondary acts performed upon the ongoing non-reflective stream of primary emotive experience” (Hochschild, 1979, p. 552). In her now classic studies on the social regulation of emotion, she focuses on the second option and takes a two-factor perspective. She coins the “secondary acts” that are performed on primary emotive experience “emotion work” (Hochschild, 1979). Originally developed in an investigation of the emotional demands of service-sector employes, emotion work corresponds to the “act of trying to change in degree or quality an emotion or feeling” or simply “to ‘work on’ an emotion” (Hochschild, 1979, p. 561). It is closely related to – and in fact an extension of – Goffman’s ideas on *The Presentation of Self in Everyday Life* (Goffman, 1959). As such, her account is strongly influenced by the principles of symbolic interactionism.

Hochschild assumes that emotion work in principle serves two goals: to either evoke or to suppress an emotion. Her account of the processes and mechanisms underlying emotion work is inspired by the ways in which professional actors evoke and shape emotions, and she makes explicit reference to Stanislavski’s *method acting* paradigm (Hochschild, 1983) to distinguish two types of emotion regulation: “deep acting” and “surface acting” (Hochschild, 1983, p. 48). Here, “deep acting” is mainly used synonymously to “emotion work,” meaning the management of a feeling or an emotional state, whereas “surface acting” is limited to modulating only the behavioral expression of an emotion. Surface acting thus equals Goffman’s (1959) description of impression management in social interactions.

Empirical research has almost exclusively focused on one specific instance on emotion work, namely “emotional labor.” Emotional labor denotes emotion work that is performed in organizational and economic contexts. It does not primarily pursue individual goals, but is rather seen as an instrumental strategy to increase economic success of an organization. Hochschild’s classic study on emotional labor of flight attendants and employes in debt collection agencies provided an empirical illustration of the concept (Hochschild, 1983, p. 89–161) as does a body of more recent studies in the sociology and psychology of work and organization (e.g., Brief and Weiss, 2002; Fineman, 2003).

## Emotion Norms

In contrast to much of psychological research on emotion regulation (but note the more recent studies mentioned above), the concept of emotion work does not primarily rely on individual norms and standards. Rather, its point of reference are socially shared (albeit at times latent) norms and rules that govern regulation. In analogy to “display rules” (Ekman, 1972, p. 225), Hochschild terms these socially shared norms directed at emotional experience *feeling rules*. A feeling rule “delineates a zone within which one has permission to be free of worry, guilt, or shame with regard to the situated feeling” (Hochschild, 1979, p. 565). These rules specify which emotions are regarded as appropriate and expected in particular situations. Based on this understanding, feelings rules are a subset of *prescriptive* social norms that indicate what “ought or ought not to be the case” under specific circumstances (Opp, 2002, p. 132). More specifically, these norms demarcate the intensity, direction, duration, and objects of emotions appropriate in a situation (Hochschild, 1979; Thoits, 2004).

Feelings rules thus are presumed to guide emotion regulation much in the same way as other social norms guide behavior. Although the coercive and compelling nature of social norms is a matter of debate, the desire for social conformity, maintenance of cooperation, circumvention of material sanctions or social exclusion, and averting negative emotions such as shame and embarrassment are amongst the most frequently mentioned reasons for emotion regulation (e.g., Bicchieri, 2006; von Scheve, 2010). Hochschild notes that feeling rules are effective in principle in two ways: as individual expectations of how we (and probably others) usually or “normally” feel in a specific situation (e.g., we expect to feel bored during the lecture of a certain colleague) or as social expectations how we *should* feel in this situation (probably excited; Hochschild, 1979). This view offers striking parallels to how social norms are conceptualized in social philosophy and psychology. The first is quite similar to the concept of *descriptive* norms, i.e., norms resulting from the perceptions of what most others (including the self) actually and usually do (Cialdini, 2007). This is how recurrent individual experiences solidify into emotional norms or conventions. Individuals develop expectations about emotions based on their own experiences and experiences of others (Thoits, 2004, p. 363). The second view indicates the existence of an *injunctive* norm that prescribes a certain kind of behavior in a specific situation (ibid.). Although conceptually related, the first is based on social information and the second is based on social evaluation, both of which can equally be applied to feeling rules.

Importantly, feeling rules are conceived of as elements of an overarching ideology, a broader system of normative social order. In the same way as normative orders guide all sorts of behaviors through norms and values, for example fairness, reciprocity, or generalized trust, they guide emotions and their expression. Whereas the concept of “ideal affect” (Tsai, 2007) and the culture-specific values ascribed to different emotions are usually *not* situation-specific, feelings rules are closely tied to specific social situations. A defining criterion of values and also of moral norms is their universality within a society and across situations (Turiel, 1983). If I value freedom, honesty, and fairness, I do so regardless of a specific situation. The same can be said about certain emotions: for example, we do not value envy or rage in most modern western societies, mostly regardless of the situation. In contrast, feeling rules as instances of social norms are bound to specific situations. We are supposed to feel sad at funerals and happy on New Year’s Eve.

Thus, from the perspective of emotion work, the social dimension of regulation not only stems from the social sharing of feeling rules, but also from mechanisms that establish links between feeling rules and (classes of) social situations. In sociology, these mechanisms are realized by “framing rules.” Such rules govern the ways in which “we ascribe definitions or meanings to situations” (Hochschild, 1979, p. 566), for example “this conversation is just a friendly chat” vs. “this conversation is already part of a job interview.” These rules for defining situations, based on certain situational cues or components, imply the validity of situation-specific feeling rules (and other social norms). These ideas have already been spelled out by symbolic interactionism, for example in Goffman’s (1974) *Frame Analysis*, and by the sociology of knowledge, in particular Berger’s and Luckmann’s (1966) treatise on *The Social Construction of Reality*. More recently, psychological research on social and socially situated cognition has illuminated the processes and mechanisms underlying framing rules (Kunda, 1999; Bless et al., 2004). This work emphasizes the automaticity and rapidity with which individuals categorize situations according to certain perceptual cues and the fundamental impact of automatic categorizations on behavior, for instance in view of stereotype activation, person perception, and emotion (e.g., Macrae and Bodenhausen, 2000; Smith and Semin, 2004, 2006; Griffiths and Scarantino, 2009).

Importantly, framing rules and the validity of feeling rules not only depend on situational information, but also on ascribed and achieved characteristics (e.g., social roles, status, power, gender, age) relative to the situation at hand. For example, a mother and her adolescent child in a social encounter with a third person will frame the situation differently based on, for example, their age and social roles. A mother might frame the situation as an insulting one and feel justified in feeling embarrassed, whereas the child might frame the situation as a joke and feel justified in feeling amused. Likewise, an encounter between a superordinate and an employe might carry framings that allow humor for the higher status individual and preclude humorous behavior for the subordinate. In institutionalized contexts, framing and feeling rules differ for customers and employes, as demonstrated by Hochschild’s (1983) classical study. This way, societies are threaded with normative orders that lead to socially differentiated patterns of emotion work.

Feeling rules not only shape emotions but also reflect the dominant views of emotion, their relative importance, and the socially accepted ways of dealing with them. Thus – in addition to valued feelings – they play a crucial role in shaping the “emotional culture” of a society, which Thoits (2004) defines as “beliefs about the nature, causes, distributions, value, and dynamics of emotions in general as well as of specific feelings” (p. 362). Social psychological research has demonstrated links between emotion cultures and social behavior in various domains (e.g., Nisbett and Cohen, 1996; Ijzerman et al., 2007; Ijzerman and Cohen, 2011). Likewise, social historical scholarship has revealed links between changing norms and values and the emotion culture of a society (Thoits, 2004, p. 360f; Cancian and Gordon, 1988; Stearns, 1993; Illouz, 1997; Reddy, 2001).

Moreover, studies in emotion work and feeling rules often adopt a “critical” stance because of the potential social, psychological, and physiological consequences of emotion work (cf. also Gross, 2002). It is frequently assumed that feeling rules create a tense relationship between socially expected emotions and actually experienced emotions. This tension gives rise to “emotional dissonance” or “emotional deviance” (Hochschild, 1983; Thoits, 1990; Jansz and Timmers, 2002), which has to be eased by means of emotion work. In the long run, the constant need for emotion work is supposed to lead to the “alienation” from one’s own feelings (Hochschild, 1983).

## Emotion Regulation and Emotion Work: Two Sides of the Same Coin?

Although many sociological and social science inquiries into emotion work are not primarily concerned with the individual components of emotion regulation, but rather with its ideological, organizational, and economic contexts (often in the sense of social criticism), there are far-reaching parallels with psychological models of emotion regulation. Investigating these parallels may not only advance our understanding of the principles of emotion work and emotion regulation, but will allow us to better (a) estimate and predict the individual consequences of emotion work in social institutional settings, (b) delineate the social and cultural embeddedness of emotion regulation, and (c) apprehend the systematic social shaping of emotion. Some of these linkages and conceptual overlaps have been described by Grandey (2000), but with an emphasis on emotional labor and organizational settings. Grandey highlights similarities between Hochschild’s (1983) concepts of deep and surface acting in emotional labor on the one hand and Gross’s (1999) process model of emotion regulation on the other hand. She uses this integrative view to develop a model of emotional labor that profits from an in-depth consideration of organizational processes and the demands of paid work (as outlined by the sociology and psychology of work and organizations) as well as form detailed accounts of situational cues, individual processes, and long-term consequences of emotion regulation. Here, I will re-iterate several of her points, but instead of focusing on organizations and emotional labor aim at a more general and “large-scale” approach at understanding the social embeddedness of emotion regulation.

**Figure 2:**
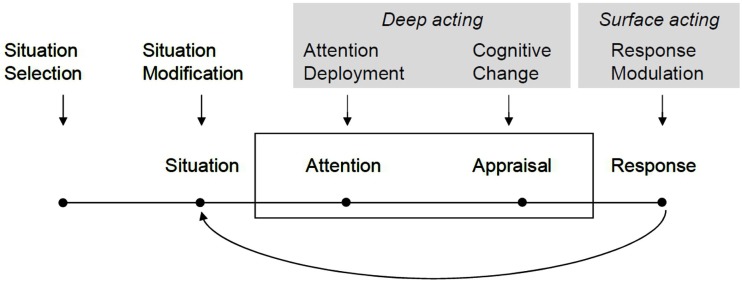
**Deep acting and surface acting in Gross’s process model of emotion regulation**. Based on Gross and Barrett (2011, p. 12).

Process models of emotion regulation give insights into the various distinct stages of emotion regulation and regulatory processes in relation to different junctures in the phases of emotion elicitation and the components of an emotion or emotion episode. I therefore start with the basic assumptions of Gross’s (1999) process model of emotion regulation illustrated above and use Hochschild’s (1979) account of emotion work to locate and specify the social and cultural determinants of emotion regulation within and on top of this model. I will also draw on other theories in the sociology of emotion to further extend Gross’s model in view of the social distribution of resources that are necessary to implement certain strategies of regulation. Figure 2 illustrates the way in which *deep acting* and *surface acting* can be understood as parts of the emotion regulatory process.

The emotion antecedent strategies of attentional deployment and cognitive change – or reappraisal – largely correspond to Hochschild’s concept of deep acting or emotion work in a narrower sense (Hochschild, 1979; Grandey, 2000). Because Hochschild’s work has a focus on emotional labor in organizational settings, it seems obvious that she emphasizes these cognitive regulation processes over situation selection and modification, mostly because employes are limited in their capabilities to select and modify situations. According to Hochschild, emotion work may consist of three elements: cognitive, bodily, and expressive. We will deal with the bodily and expressive components later and focus on the cognitive element. Cognitive strategies in models of emotion work refer to attempts to “change images, ideas, or thoughts in the service of changing the feelings associated with them” (Hochschild, 1979, p. 562).

Most interestingly, although hidden in a footnote, Hochschild (ibid.) explicitly relates these cognitive strategies to appraisal theories of emotion, in particular Lazarus’s (1966) approach, which are also foundational to process models of emotion regulation. However, emotion work is only seldom seen in this light of appraisal theory. It can be understood as an attempt at “recodifying” situations or at reclassifying them into “previously established mental categories” (ibid.). This deliberate and conscious recodification (reappraWisal) acts upon previous automatic codifications and interpretations (appraisals) that gave rise to the initial emotion.

Response modulation in Gross’s process model resembles the idea of surface acting in theories of emotion work. Here, Hochschild’s (1979, p. 562) ideas of regulating the bodily, i.e., physiological, components, or “symptoms” of emotions (e.g., respiratory control) are in line with Gross’s view of response-oriented regulation. The same holds for the expressive components which are, strictly speaking, a class of bodily reactions. Importantly, and in contrast to the process model of regulation, Hochschild is interested in bodily and expressive regulation primarily in view of the their effects on the regulation of the underlying feeling, for example trying to smile not only for “interactive” reasons, but also to change the phenomenal feeling (ibid.). In line with Gross, she acknowledges that antecedent- and response-oriented strategies often go hand in hand.

**Figure 3:**
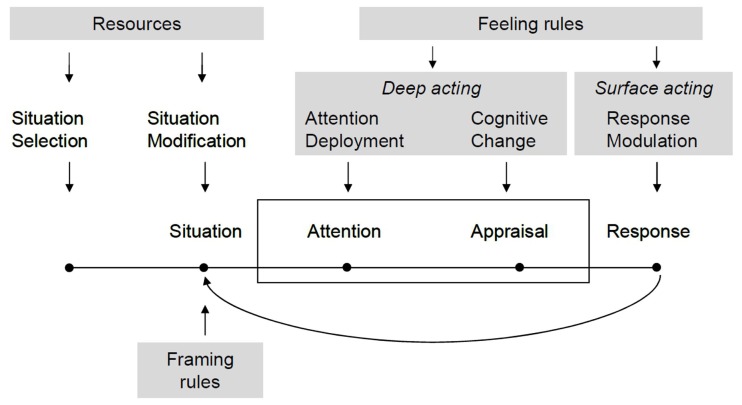
**The influence of resources, framing rules, and feeling rules on emotion regulation**. Based on Gross and Barrett (2011, p. 12).

Importantly, in uncovering the social determinants of emotion regulation, both strategies have to be linked to certain norms and values that serve as emotion regulatory goals, in particular to the feeling rules outlined above. The concept of feeling rules (or emotion norms, more generally) is an important addition to the process model, because it is highly situation-specific. Whereas accounts of emotion regulation that emphasize cultural values as emotion regulation goals take a more universal approach (e.g., Tsai, 2007), feeling rules presuppose situation-specific framing rules indicating their validity. As shown in Figure 3, feeling rules primarily inform deep and surface acting or attentional deployment, cognitive change, and response modulation.

Feeling rules are less suited to apply to regulatory strategies that aim at situation selection or modification because they are situation-specific. This is why their influence on emotion regulation is focused on deep and surface acting in this model. By accounting for the influence of feeling rules, the process model of regulation can accommodate emotion regulatory goals that are socially shared, highly interactive, and situation-specific, and at the same time systematically evoked in accordance with institutional settings and corresponding framing rules and social cognitive processes of situation perception.

Therefore, the significance of framing rules in constituting the social dimension of regulation is closely linked to situation selection and situation modification strategies. Given that individuals are able to actively seek or avoid situations or change certain parameters of an existing situation, they simultaneously alter the framing rules that are associated with a situation. Selecting or modifying situations usually results in different frames that are applied and different rules that go along with the new or modified situation. These in turn imply changes in situation-specific feeling rules in such a way that they are more compatible with the regulatory goals an individual actively pursues.

Importantly, framing rules and associated feeling rules are not given by nature or stand firmly without alternatives. Hochschild (1979, p. 566) emphasizes that they have an “ideological stance,” that they are in fact the “bottom-side” of ideology. Conceiving of ideologies more broadly as the major competing cultural systems of meaning making, this means that there are always alternatives as to how a prevailing situation is to be framed according to which ideological stance. For example, feminist or gender mainstreaming proponents in a committee meeting will probably apply different sets of framing rules than, say, the very conservative representatives. Thus, framing rules as well as associated feeling rules always reflect a particular order of sense-making that is prevalent in a social institutional setting. However, changing the framing rules for specific situations is not an easy task. Although they may have an ideological and “socially constructed” background, framing rules become deeply embodied and ingrained into how we perceive the world that they are hardly alterable voluntarily and on a moment-to-moment basis.

Moreover, feeling rules are not only situations-specific, but their validity also depends on the individuals involved in a situation, in particular on their social roles and status positions and related social categories. For example, research has aptly documented the different feeling rules that are in place in one and the same situation for men and women (Cancian and Gordon, 1988; Brody and Hall, 2000; Simon and Nath, 2004) or people of different age (von Salisch, 2001; Hepworth, 2007). This systematic distribution of feeling rules across the social spectrum (both, vertically and horizontally) should thus lead to marked differences in emotion regulatory behavior across social groups and institutional settings.

Finally, as indicated in Figure 3, the possibilities for antecedent regulation, in particular for situation selection and modification, depend on individuals’ capacities to actually select and change a situation. These capacities are constrained by several factors, in particular the institutional setting and available resources. Some institutional settings such as third-sector employment with frequent customer contact leave only little room for selecting situations at will. Also, situation selection aiming at emotion regulation in certain areas of the family or in educational settings may be hard to achieve. As a general rule, the more formalized an institutional setting is and the more individuals are bound to a specific social role, the less likely becomes situation selection as a strategy of emotion regulation.

Moreover, selecting and modifying situations requires adequate resources to do so. This includes cultural resources in the broadest sense, such as knowledge on how to change or select a situation; it may require economic resources as a means to actually implement selection or modification, and this strategy may also need the adequate social resources, in particular status and power (e.g., Kemper, 1978), that enable individuals vis-à-vis others to change a situation. Importantly, as social science research has repeatedly documented over the past decades, these resources are not arbitrarily distributed in society, but highly inter-correlated and associated with social structure (e.g., Massey, 2008). Systematic social differences in the available resources to implement certain strategies of emotion regulation should thus – in conjunction with norms and regulatory goals – lead to discernable social patterns in emotion regulation.

## Discussion

In this article I have outlined an approach to understanding the social dimension of emotion regulation by integrating micro-level process models and the concepts of emotion work and feeling rules. From a sociological perspective, two-factor process models offer insights into the regulation of emotion that are closely linked to the processes of emotion elicitation and the immediate situational context of an emotion episode. Understanding the broader and longer-term “regulation” of emotion, as demanded by one-factor models, is more effectively accomplished by other paradigms in the sociology and psychology of emotion, such as social structural and cultural approaches (e.g., von Scheve and von Luede, 2005; Boiger and Mesquita, 2012). On the other hand, psychological process models profit from consideration of ways to incorporate the social and cultural embeddedness of regulation, as is already done in works highlighting the role of emotion values (e.g., Tamir, 2009).

In addition to these works, the various linkages discussed herein highlight situationally specific social and cultural parameters of emotion regulation. Using the concepts of deep acting and surface acting, I have outlined the ways in which feeling rules as specific instances of a broader class of emotion norms (including, for example, display rules) serve as emotion regulatory goals reflecting (injunctive) social expectations and (descriptive) personal standards. I have also highlighted the importance of framing rules which link situational context to the validity of specific feeling rules. It has become clear that emotion regulation in social contexts is also fundamentally dependent on prevailing “ideologies” or prevalent systems of meaning making that may differ across groups and categories of individuals. Finally, I have emphasized that the emotion antecedent strategies of situation selection and modification strongly depend on available resources, which in turn are systematically and unequally distributed in a society.

### Consequences of emotional labor and emotion regulation

This specification of process models of emotion regulation may, for example, help in achieving a better understanding of the individual and social consequences of emotion regulation, a critically debated topic in sociology. Hochschild (1983), for example, has expressed concerns about the alienation from one’s own feelings and the psychological and physiological strains that go along with emotional labor (see also Grandey, 2000). In this regard, studies on the consequences of emotion regulation have revealed significant differences between deep acting and surface acting and between antecedent- versus response-oriented regulation (Gross, 2002). If, in fact, theoretical assumptions made by models of emotion regulation concur sufficiently with those of emotion work and emotional labor, insights from existing research might aide in clarifying the actual consequences of emotional labor. Conversely, and based on the conjecture that emotion regulation in private and organizational settings are fundamentally different from one another – based on the corresponding situational framing rules – empirical studies could tap into these differences and, for instance, account for the situational context (private vs. organizational) as a moderating variable in assessing the psychological and physiological consequences of emotion work and emotion regulation.

### Intra-societal variation in emotion regulation

Given the existing studies on cultural differences in emotion regulation (e.g., Mauss et al., 2008), the integrative model developed here may to help to investigate systematic differences in emotion regulation *within* societies. Sociology is classically concerned with examining social differentiation at various levels. One (vertical) approach is to conceive of differentiation as stratification and to look at the unequal distribution of and access to resources across society, for instance in different social classes. Another (horizontal) approach is to investigate social differentiation based on different tastes and preferences, as is evident in different lifestyles. Bourdieu (1984) has famously offered and account of linking both perspectives using the concept of cultural capital. Recently, there is an increased interest in these linkages in social psychology. Studies have demonstrated ways in which “class culture” impacts behavior, including emotion. For example, Piff et al. (2010) have shown that social class systematically influences prosocial behavior (see also Kraus and Stephens, 2012) and Rackow et al. (2012) show how social inequality is related to the frequency of experiencing anger and anxiety. In this vein, the proposed model may help in understanding the emotion culture – and its constitutive feeling and framing rules – of social classes and the emotion-related tastes and preferences of certain lifestyles. Just like Bernstein (1971) theorized on restricted vs. elaborated codes of language use in lower and upper classes, classes could be characterized by different patterns of emotion regulation. Empirical studies can investigate whether such differences exist at all and how they are brought about, for example by differences in feeling rules or the resources that allow for situation selection and modification in emotion regulation.

### The social and cultural shaping of emotion

Finally, the extended model developed in this article may provide new insights into the long-term cultural shaping of emotion. If individuals are required to adapt their emotions to prevailing feeling rules as instances of ideologies or “emotion regimes” (Reddy, 2001), then situation-specific emotion regulation is a process that clearly contributes to this shaping. Much has been speculated on the role of social norms and practices in the culture-specific shaping of emotions. Many of these macro- or discourse-level approaches fall short of recognizing that emotions are also fundamentally psychological and bodily phenomena and seldom provide elaborated models of how to link culture, cognition, and emotion in an integrative framework. A model of emotion regulation that accounts for both, the social influences and the psychological mechanisms through which these influences are mediated can enhance our understanding of how exactly culture and society shape emotion. In conjunction with the existing one-factor approaches to emotion regulation (e.g., Kappas, 2011), two-factor models that decidedly consider individuals’ embeddedness into culture and social structure are instructive, for example, in empirically investigating differences between adaptive processes of socialization and internalization in relation to general emotion values and those based on explicit and situation-specific normative obligations.

## Author Note

Christian von Scheve, Department of Sociology and Cluster of Excellence “Languages of Emotion,” Freie Universität Berlin, Berlin, Germany. Supported by the Cluster of Excellence “Languages of Emotion,” Freie Universität Berlin, within the Excellence Initiative of the German Research Foundation (DFG).

## Conflict of Interest Statement

The author declares that the research was conducted in the absence of any commercial or financial relationships that could be construed as a potential conflict of interest.
